# Zipper repair of 3 cm iatrogenic colonic perforation with dual‐channel endoscope and twin grasper‐assisted titanium clip

**DOI:** 10.1002/deo2.276

**Published:** 2023-07-21

**Authors:** Wei Yang, Wei Wen, Xiaoli Ren, Qi Zhai, Shupei Li, Ji Xuan

**Affiliations:** ^1^ Department of Gastroenterology Jinling Clinical Medical College Nanjing Medical University Nanjing China; ^2^ Department of Gastroenterology The Second Affiliated Hospital of Nanjing University of Chinese Medicine Nanjing China; ^3^ Department of Gastroenterology Jinling Hospital Affiliated to Nanjing University Nanjing China; ^4^ Department of Gastroenterology Jinling Clinical Medical College Nanjing University of Chinese Medicine Nanjing China; ^5^ Department of Gastroenterology Jinling Hospital Affiliated Hospital of Medical School Nanjing University Nanjing China

**Keywords:** clips, colonoscopy, intestinal perforation, tantalum

## Abstract

A 53‐year‐old man undergoing painless colonoscopy for long‐term diarrhea. After colonoscopy withdrawing to the sigmoid colon, a local perforation was found, about 3 cm in size, oval in shape. We used a two‐channel endoscope and grip to pull the edges of the intestinal wall on both sides of the perforation site to close together, and then repair the 3 cm oval perforation of the colon through multiple ordinary titanium clips. The patient had no obvious infection after surgery and recovered well after 1 month of follow‐up. Preliminary experience has shown that using multiple titanium clips under dual‐channel endoscope to zipper suture can effectively repair 3 cm iatrogenic colonic perforations.

## INTRODUCTION

In recent years, endoscopic repair of iatrogenic colonic perforation has been widely used in clinical practice, and it has been confirmed that it has a high success rate and good safety. It is widely believed that through‐the‐scope clip (TTSC) should be given priority for perforations smaller than 1cm, while over‐the‐scope clip (OTSC) or combined purse‐string suture techniques should be considered for larger perforations.^1^, ^2^, ^3^, ^4^ Here, for the first time, we have successfully applied a new suture technique to the clinic, which has been confirmed to repair 3 cm perforations in size by TTSC under a dual‐channel endoscope.

## CASE REPORT

A 53‐year‐old man underwent a painless colonoscopy (Dezocine analgesia and propofol for induction and maintenance of anesthesia) because of long‐term diarrhea. Under the endoscope, the mucosa of the sigmoid colon was congestive, edematous, and angled locally. After colonoscopy withdrawing to the sigmoid colon, a local perforation was found, about 3cm in size, oval in shape, and no active bleeding. Therefore, endoscopic treatment of the perforation was considered immediately. We used a dual‐channel endoscope and multiple titanium clips to perform zipper anastomosis at the perforation. After the operation, the wound was clean and tidy without blood osmosis. The wound was rinsed with iodophor, and a drainage tube was placed on the anal side, which was drawn out through the anus and fixed.

**FIGURE 1:**
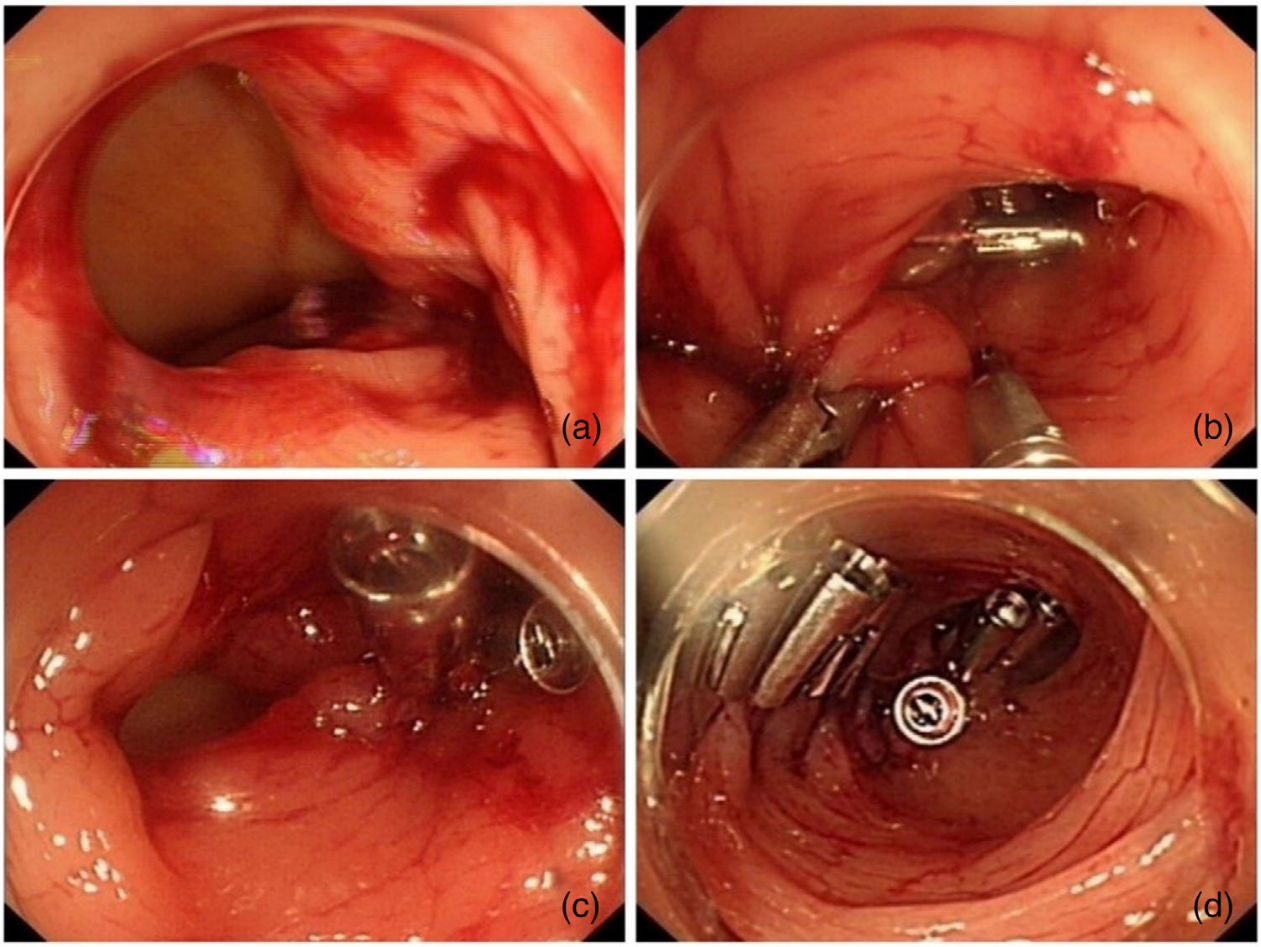
Zipper repair of colonic perforation with multiple titanium clips under endoscope. (a) An oval perforation about 3 cm in the sigmoid colon. (b) Repair perforation with over‐the‐scope clip endoscopic clamp‐assisted anastomosis and titanium clips in a dual‐channel endoscope. (c) Partially repaired perforation. (d) Zipper repair completed.

**FIGURE 2:**
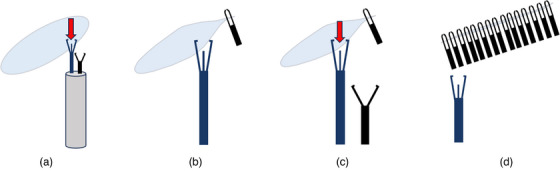
Schematic diagram of zipper repairing colonic perforation with a dual‐channel endoscope and multiple titanium clips. (a) In one channel, use over‐the‐scope clip Twin Grasper to grasp one edge of the perforation and bring it close to the opposite edge. (b) Place the first titanium clip to seal the perforation. (c) Repeat step A and prepare to place the second titanium clip to seal the perforation. (d) Repeatedly place multiple titanium clips until the zipper repair process is completed.

The specific repair methods are as follows: (1) Use a dual‐channel endoscope (Olympus GIF‐2TQ260M) to reach the perforation site. (2) In one channel, use OTSC Twin Grasper (OVESCO, Germany) to grasp one edge of the perforation and bring it close to the opposite edge. (3) In another channel, use titanium clips (Nanjing minimally invasive, ROCC‐D‐26‐195‐C, opening diameter of ≥10 mm, working length of 1950 mm) to clamp the edges on both sides. (4) Repeat until the entire perforation is completely closed with 15 titanium clips. Our experienced endoscopists have about 20 years of endoscopic experience and are proficient in most types of endoscopic surgery. The gas injection during operation is CO_2_ gas, and the procedure time is about 35 min. (The endoscopic process is shown in Figure 1 and the operation diagram is shown in Figure 2)

The patient had a history of intestinal polyps. On the day after colonic perforation repair, the patient had tolerable abdominal pain and discomfort without fever. We used cefoperazone sodium sulbactam sodium and levonidazole for anti‐infection, pantoprazole to inhibit acid for stomach protection, and snake venom hemagglutinase to prevent bleeding. The patient was given fasting, and the fat milk glucosamine was used for his nutritional support. His highest temperature was measured at 37.3°C on the same day. On the 2nd day after the operation, the patient indicated that the abdominal pain was relieved, less drainage was found in the drainage tube, and part of the bile‐like fluid could be drained after adjustment. The body temperature was 36.6°C, the white blood cell count was 15.5 × 10^9^/L, the percentage of neutrophils was 94.30%, and the hypersensitive C‐reactive protein was 126.7 mg/L. On the 5th day, the patient's abdominal pain gradually improved, the drainage tube drained bile‐like fluid 50 ml, the body temperature was 36.2°C, the white blood cell count was 5.5 × 10^9^/L, the percentage of neutrophils was 79.50%, hypersensitive C‐reactive protein was 79.5 mg/L and procalcitonin were normal, so somatostatin was discontinued. On the 8th day, the abdominal pain disappeared, so the use of antibiotics was stopped. On the 10th day, the patient resumed a liquid diet. After 1 month follow‐up, the patient's condition remained stable without abdominal pain and distension, normal stool color, no fever, and other discomfort.

## DISCUSSION

Different from the thermal/electrical injury during colonoscopy, the perforation in this patient during colonoscopy is mainly caused by direct mechanical injury of an endoscope or pneumatic injury caused by gas injection, so the perforation is often larger.^1^ For large colonic perforations, endoscopic repair experience is less, but according to a few reports, it is still safe and effective,^2^, ^5^ and our practice has also proved that endoscopic repair of large colonic perforation is feasible. At present, there are two main methods for endoscopic repair of large mucosal defects, one is to use OTSC and the other is to use TTSC combined purse‐string suture techniques. The use of OTSC closure perforation is more mainstream and the closure is firm, but OTSC is more expensive and requires the endoscopy center to introduce OTSC. In particular, many of our primary hospitals have not yet introduced OTSC. At the same time, we also noticed that although OTSC is tightly sutured, it is not easy to fall off, and long‐term retention in the tissue can be regarded as a foreign body. TTSC combined with purse‐string suture techniques can also repair colon perforation very well, we have tried to use this method, but the postoperative patient did not heal well, and eventually underwent open surgery for salvage treatment, we speculated that the intestinal wall tension on the long axis is excessive during the perforation of the long colon with purse sutures, so healing is poor.

Referring to past experience,^6^, ^7^, ^8^ we have successfully applied a new technology to patients for the first time, that is, using multiple ordinary titanium clips can successfully repair 3cm‐sized oval colonic perforation. The feasibility of this method has been initially demonstrated in their animal tests. We use multiple titanium clips to close the perforation, because the titanium clip mainly clamps the mucosal layer, so the titanium clip can generally automatically fall off after the tissue heals. This method is not difficult to operate. We use a dual‐channel endoscope and grip to pull the edges of the intestinal wall on both sides of the perforation site close to each other, so as to make up for the lack of TTSC arm span,^9^ and then form a zipper suture with multiple titanium clips. This method is also flexible and does not require a special repair device, and can effectively reduce the tension on the long axis during long perforation suture, which is helpful for wound healing and is more suitable for long perforation, which can provide a new idea for the clinical repair of large iatrogenic colonic perforation. It is especially suitable for some medical units that have not yet introduced OTSC. However, this method is less feasible in the proximal colon, and blind use may have a high safety risk (surgical remediation may even be required), so this method may be more suitable for the distal colon. At the same time, we also need to note that this method uses more clips(but is still far cheaper than OTSC), and the endoscope needs to be replaced by the dual‐channel endoscope, which not only increases the operation time and gas injection but also puts forward requirements for the operator and the equipment preparation of the endoscope center. However, the presence of gas in the abdominal cavity is not the indication of operation, abdominal pain and fever are the most important factors to determine whether to operate.^10^ We observed that the patient in this case recovered well after the operation and there was no indication of an operation.

## CONFLICT OF INTEREST STATEMENT

None.
